# Changes in Emergency Department Care Intensity from 2007–16: Analysis of the National Hospital Ambulatory Medical Care Survey

**DOI:** 10.5811/westjem.2019.10.43497

**Published:** 2020-02-21

**Authors:** Shih-Chuan Chou, Olesya Baker, Jeremiah D. Schuur

**Affiliations:** * Brigham and Women’s Hospital, Department of Emergency Medicine, Boston, Massachusetts; † The Warren Alpert Medical School of Brown University, Department of Emergency Medicine, Providence, Rhode Island

## Abstract

**Introduction:**

Emergency departments (ED) in the United States (US) have increasingly taken the central role for the expedited diagnosis and treatment of acute episodic illnesses and exacerbations of chronic diseases, allowing outpatient management to be possible for many conditions that traditionally required hospitalization and inpatient care. The goal of this analysis was to examine the changes in ED care intensity in this context through the changes in ED patient population and ED care provided.

**Methods:**

We analyzed the National Hospital Ambulatory Medical Care Survey (NHAMCS) from 2007–2016. Incorporating survey design and weight, we calculated the changes in ED patient characteristics and ED care provided between 2007 and 2016. We also calculated changes in the proportion of visits with low-severity illnesses that may be safely managed at alternative settings. Lastly, we compared ED care received and final ED dispositions by calculating adjusted relative risk (aRR) comparing ED visits in 2007 to 2016, using survey weighted multivariable logistic regression.

**Results:**

NHAMCS included 35,490 visits in 2007 and 19,467 visits in 2016, representing 117 million and 146 million ED visits, respectively. Between 2007 and 2016, there was an increase in the proportion of ED patients aged 45–64 (21.0% to 23.6%) and 65–74 (5.9% to 7.5%), while visits with low-severity illnesses decreased from 37.3% to 30.4%. There was a substantial increase in the proportion of Medicaid patients (22.2% to 34.0%) with corresponding decline in the privately insured (36.2% to 28.3%) and the uninsured (15.4% to 8.6%) patients. After adjusting for patient and visit characteristics, there was an increase in the utilization of advanced imaging (aRR 1.29; 95% confidence interval [CI], 1.17–1.41), blood tests (aRR 1.16; 95% CI, 1.10–1.22), urinalysis (aRR 1.22; 95% CI, 1.13–1.31), and visits where the patient received four or more medications (aRR 2.17; 95% CI, 1.88–2.46). Lastly, adjusted hospitalization rates declined (aRR 0.74; 95% CI, 0.64–0.84) while adjusted discharge rates increased (aRR 1.06; 95%CI 1.03–1.08).

**Conclusion:**

From 2007 to 2016, ED care intensity appears to have increased modestly, including aging of patient population, increased illness severity, and increased resources utilization. The role of increased care intensity in the decline of ED hospitalization rate requires further study.

## INTRODUCTION

Emergency departments (ED) have become the center for acute, episodic care in the United States (US) over the past two decades. The growth in visit volumes to EDs across the nation has exceeded population growth,^1^,^2^ despite concurrent ED closures.^3^ A rising proportion of hospital admissions are originating from the ED.^4^,^5^ These changes have propelled EDs to significantly expand its diagnostic and treatment capabilities, most notably the availability of advanced imaging and observation unit care.^6^,^7^ These changes allowed EDs to take a larger role in acute care delivery and increased the intensity of care delivered in EDs over time. Evidence from the early 2000s showed a rapid rise in advanced imaging use as well as an increase in laboratory testing and treatment utilization.^6^,^8^,^9^ However, these studies coincided with the proliferation of advanced imaging technology and outpatient care pathways.^6^,^10^ Whether these trends of rising care intensity and utilization continued beyond the initial expansion is unclear.

While the demand for emergency care and ED capabilities continues to expand, the rising healthcare expenditure has led policymakers and clinical leaders to implement cost reduction policies, aiming to decrease low-value care and avoidable hospitalizations. On the one hand, efforts to decrease low-value care, such as the formation of Choosing Wisely guidelines by the American College of Emergency Physicians, may lower care intensity through decreased avoidable testing and treatment use. On the other hand, to reduce avoidable hospitalizations, ED care intensity may increase so that EDs may facilitate lower-cost outpatient management or ED-based observation care for conditions that conventionally have required hospitalized care.^10^ Therefore, the net change in emergency care intensity as a result of efforts in low-value care reduction and the shift toward outpatient care remains unknown and warrants an updated evaluation.

The goal of this study was to use a nationally representative dataset to assess the changes in the intensity of the care provided in US EDs over the past decade. We examined the changes in the complexity of the ED patient population and the services provided in US EDs between 2007 and 2016.

## METHODS

### Dataset

We analyzed the 2007–2016 public-use datasets of the National Hospital Ambulatory Medical Care Survey (NHAMCS) ED sample. NHAMCS is an annual survey conducted by the Ambulatory and Hospital Care Statistics Branch of the National Center for Health Statistics (NCHS). The NHAMCS consists of multistage, probability samples of visits to hospital-based EDs in the US. Each encounter was assigned a weight and corresponding design variables to generate nationally representative estimates and standard errors. Detailed sampling and survey methodologies are available on the NCHS website.^11^ This study was exempt from review by the institutional review boards of the authors’ institutions.

### Patient Characteristics

We first examined demographic characteristics of ED patients, including age groups, gender, race/ethnicity, and insurance status, to explore the change in patient complexity as a contributor of changing ED care intensity. Of note, NCHS imputed approximately 20–25% of visits where race (Black, White, and other groups), ethnicity (Hispanic and non-Hispanic), or both were missing in each survey year. We used the two variables to categorize patients into four racial/ethnic groups: non-Hispanic White, non-Hispanic Black, Hispanic, and others. For insurance status, we used the variable *paytyper*, which categorized patients into a hierarchy by the primary insurance that is providing the patient coverage, including private insurance, Medicare, Medicaid, other insurance, and uninsured. We accounted for the changes in the hierarchy used to construct this variable in year 2007 where Medicare and Medicaid dual-eligible patients were categorized as Medicaid, whereas they were categorized as Medicare in the remainder of the survey years included in this study.^8^

**Population Health Research Capsule**

What do we already know about this issue?

Emergency departments (ED) occupy a more central role in acute, unscheduled care by providing an increasing proportion of acute care. EDs are also a rising source of hospital admission.

What was the research question?

Is emergency care rising in intensity, as defined by increased patient complexity, testing, and treatment?

What was the major finding of the study?

From 2007–2016, ED patients have become more complex. Diagnostic and treatment use continued to rise, but admission rates have declined.

How does this improve population health?

Future research should examine whether increased ED care intensity has directly improved the value of care, which will inform future delivery and payment system reform for emergency care.

We included other visits characteristics including region (Northeast, Midwest, South, and West) and whether the care team included any physician assistants, nurse practitioners, or residents. We also included time of visit, categorizing visits into weekday (8 am to 5 pm Monday through Friday), weeknights (5 pm to 8 am starting on Monday through Thursday), and weekends (not weekdays or weeknights). To further assess the complexity of ED visits, we adopted a previously published definition to categorize ED visits as low-severity (Appendix Table A).^12^

We considered including triage severity and initial visit vitals as an additional measure of patient complexity; however, there was a significant proportion of missing values to both (approximately 20–30% and 10–15%, respectively, across survey years), which substantially limited their interpretability and validity. Therefore, we did not include triage severity or visit vitals.

### Emergency Care Delivered

We next examined the care and services delivered as a measure of ED care intensity, including advanced imaging, radiographs, blood and urine testing, electrocardiograms (ECG), and bedside procedures. Advanced imaging included a patient getting any computer tomography (CT) and magnetic resonance imaging (MRI). We also included patients who received ultrasound, since ultrasound, like CT or MRI, is often not readily available in the outpatient care setting. We categorized blood testing into routine—including complete blood counts, chemistry panels, liver function tests, coagulation studies, cardiac enzymes, alcohol level—and special testing, including blood cultures, human immunodeficiency virus testing, toxicological screening, and arterial blood gas.

We categorized bedside procedures into urgent care procedures—including orthopedic care (cast/splint), wound care (such as laceration repair and incision and drainage), urinary catheter placement, and critical care procedures—including cardiopulmonary resuscitation, and endotracheal intubation. This categorization scheme was made as patients receiving these procedures are clinically distinct although all warranting direct time from clinicians. The selection of these procedures was limited by the availability of procedural indicators throughout the study period. For example, an indicator for non-invasive positive pressure ventilation was not available until 2012.

To explore the connection of care intensity with the changes in downstream outcomes, we also examined final disposition of ED visits. We considered admission to inpatient or observation units as hospitalizations for several reasons. For two-thirds of US hospitals, observation care is delivered through inpatient floors and structured similarly to inpatient admissions.^13^,^14^ Recent evidence further suggests that observation care may be replacing traditional inpatient hospitalizations or readmissions.^15^, ^16^ Lastly, from the patient’s perspective, observation stay is likely a similar experience to hospitalized care on inpatient units.

### Statistical Analyses

We first calculated proportions of ED visits for each patient characteristic and care received, comparing 2007 to 2016 survey years. Specifically, for each patient characteristic, we calculated weighted national visit counts as well as proportions of all ED visits to illustrate both absolute change in the number of ED visit and relative changes in proportion of ED visits. We also calculated the weighted total number of annual ED patient visits for all years between 2007 and 2016 that were discharged, hospitalized (including both inpatient and observation), received advanced imaging, blood test, or four or more medications.

For ED care delivered, we calculated the unadjusted proportions of ED visits receiving each category of ED care. We further calculated the unadjusted and adjusted relative risk of receiving care in each category comparing 2016 to 2007, using survey-weighted logistic regression and *margins* post-estimation command,^17^ accounting for differences in patient characteristics, including age, gender, race, insurance status, ambulance use, region, time of visit, presence of physician assistant, nurse practitioner, or resident. We calculated 95% CI for all relative risks; however, hypothesis testing was considered significant at alpha of 0.01 for two-tailed test, in accordance to NCHS guidelines for NHAMCS 2015 and 2016.^18^

We performed all analyses and calculations of national estimates using *svy* package in Stata 15.0 (StataCorp, College Station, TX), which allowed us to incorporate the corresponding survey weights and account for complex survey design in the estimation for standard error.

## RESULTS

### Visit Patient Characteristics

From 2007 to 2016, NHAMCS sampled a total of 289,188 ED visits, with 35,490 visits in 2007 and 19,467 visits in 2016, representing 117 million ED visits in 2007 and nearly 146 million ED visits in 2016. Table 1 shows the visit patient characteristics in 2007 and 2016. Visits across all age groups increased in the total number of visits. The proportion of ED patients aged 45–64 and 65–74 slightly increased without substantial overall changes in the distribution of ED patients by age groups between 2007 and 2016.

Compared to 2007, in 2016 Medicaid visits significantly increased from 22.2% to 34.0% while there were decreases in the proportion of ED visits by uninsured (15.4% to 8.6%) and privately insured patients (36.2% to 28.3% (Table 1). Lastly, although the proportion of visits arrived by ambulance were similar, compared to 2007, the proportion of ED visits with low-severity diagnoses decreased from 37.3% to 30.4%. Notably, the weighted total number of low-severity visits increased only slightly (41.0 million visits to 41.6 million visits).

### Emergency Care Services Delivered

Table 2 shows the proportion of ED visits receiving each testing or treatment and the unadjusted and adjusted relative risk of receiving the care comparing 2016 to 2007. The proportion of ED visits that received diagnostic testing have increased slightly, including CT/ MRI (aRR 1.25; 95% CI, 1.14–1.36), basic blood tests (aRR 1.11; 95% CI, 1.05–1.17), urine tests (aRR 1.17; 95% CI, 1.09–1.26), and ECGs (aRR 1.18; 95% CI, 1.08–1.28). The proportion of ED visits receiving four or more medications during ED care increased more than two-fold (aRR 2.13; 95% CI, 1.84–2.42). In contrast, the proportion of ED visits receiving urgent care procedures decreased (aRR 0.72; 95% CI, 0.63–0.80), as well as the proportion of ED visits that led to hospitalization (aRR 0.73; 95% CI, 0.62–0.83).

**Figure 1:**
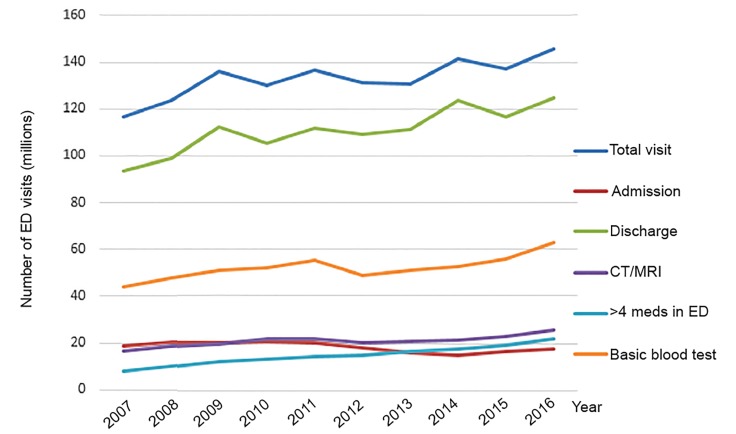
Weighted total number of emergency department (ED) visits, by care provided and disposition, National Hospital Ambulatory Medical Care survey 2007–2016. *CT*, computed tomography; *MRI*, magnetic resonance imaging.

When examined by total visit counts, the number of ED visits that led to hospitalizations remained relatively unchanged, while the increase in discharged visits parallels the upward trend in total ED visit volume (Figure 1).

## DISCUSSION

From 2007 to 2016, the total visit volume to US EDs has continued to rise while the complexity of ED patients and the intensity of emergency care delivered has grown modestly. We found that the patient population has aged slightly but the proportion of ED patients with low-severity illnesses has declined. There is also a modest increase in the utilization of testing and medication treatments. However, there was a notable decrease in the proportion of ED visits leading to hospitalizations, which appears largely driven by the increase in the number of discharged visits while the estimated number of ED hospitalizations remains largely unchanged.

Although the growth in discharged visits may suggest that the overall acuity of the ED population decreased, we instead observed that there is a modest increase in overall patient age and a decline in the proportion of ED visits with low-severity illnesses, suggesting a rise in the complexity of the ED patient population. These findings correlate with a decline in the proportion of visits receiving urgent care procedures, such as abscess drainage and orthopedic care, which are more commonly low-severity visits. Indeed, recent claims data analysis of the Nationwide Emergency Department Sample that found ED patient population is growing older with higher burdens of comorbid conditions.^19^ Likely as a result of the recent Medicaid expansion under the Affordable Care Act, we also observed a large increase in Medicaid beneficiaries and a decline in uninsured patients. As many uninsured gain coverage under Medicaid expansion and begin seeking care, previously undiscovered and untreated conditions may also contribute to the increasing complexity of the ED patient population.^20^ Taken together, US EDs are seeing an increasingly complex patient population without increasing the number of patients hospitalized.

The opposing trends of decreasing ED hospitalization but rising ED patient complexity suggest that a proportion of patients who would have likely been admitted in the past are now managed in the outpatient setting from the ED. These concurrent trends may, in part, explain the continued rise in the utilization of diagnostic testing and treatment intensity that we observed. As policymakers sought to reduce short-stay hospitalizations through policies such as the Recovery Audit Contractors program,^21^ EDs have become the center for expedited diagnosis, risk-stratification, and treatment for many conditions that traditionally warranted hospitalized care, such as chest pain, cellulitis, syncope, and transient ischemic attack. To fulfill these roles, EDs have adopted critical care pathways, which likely contributed to a rise in care intensity but reduced hospitalizations.^10^ Future studies are needed to shed light on the effect of condition-specific care pathways on care intensity and resource utilization in the ED.

The continued increase in advanced imaging use warrants attention. The rapid rise in advanced imaging in the early 2000s has led policymakers and clinical leaders to be concerned with overuse and emphasize reduction of low-value advanced imaging use.^6^,^8^,^9^ Although we observed no decrease in advanced imaging use, compared to prior studies, the increase in ED advanced imaging rates during our study period was relatively modest. A possible explanation may be that the rapidity with which advanced imaging use rose was largely due to the initial proliferation of imaging technology. As imaging technology has become ubiquitous in US EDs,^22^ the increase in advanced imaging rate has slowed down.

While the continued increase in advanced imaging use may have helped facilitate the downward trend in hospital admissions, this observation may also suggest that low-value advanced imaging remains prevalent. Examination of low-value advanced imaging among headache and syncope ED visits have shown that imaging rates increased rapidly prior to 2007.^23^ From 2007 and on, while the trend in low-value imaging use plateaued, the rate of use remained high.^23^ Future research will be needed to examine how increased advanced imaging use has influenced ED hospitalization practices.

Our results contribute to the growing literature that has documented the shifting practice of emergency care. There has been significant interest in examining the changes in ED care that may explain rising emergency care expenditures. While the volume of ED visits has grown at a pace exceeding population growth,^24^ costs per ED visit have also grown substantially.^25^ The latter likely resulted from a combination of increased cost for ED visits at the same levels of complexity and the rising proportion of visits billed at higher levels of complexity.^26^ Although, as we demonstrated, services provided during an ED visit have grown in intensity, it only partially accounts for the changes in higher complexity visit billing.^27^

Furthermore, we found that intensity has increased even after controlling for patient and visit characteristics. Together, these shifts likely result in the rising costs of emergency care; however, whether the increase per ED visit in cost reflects a corresponding increase in the value of emergency care is not known. In our study period, we found a concurrent decline in inpatient hospitalization from the ED, which leads us to hypothesize that more intense emergency care services have increased ED visit value by reducing inpatient hospitalizations. Future studies are needed to more rigorously demonstrate the association between changes in care intensity and patient outcomes and downstream resource utilization in order to assess the changes in the value of emergency care.

## LIMITATIONS

Our study is bound by the limitations of a national survey, including its cross-sectional nature as well as the potential for misclassification in patient visit characteristics, ED care provided, and diagnoses. The dataset also provided limited ability to assess the complexity of ED patients due to a significant proportion of missing data such as triage categories and presenting vital signs. As the survey changed over the years, we only selected variables such as a subset of procedures or blood tests that were present throughout the study period. NHAMCS also does not differentiate between admissions to ED observation vs observation status on an inpatient service. With the increasing prevalence of ED-based observation units, we expect there has been an increasing shift away from observation status on inpatient services.^7^

A key aspect of intensity not measured in our study was the change in physician workforce over time. Estimates from prior studies showed that the number of emergency medicine-trained physicians increased from 26,826 in 2008 to 35,856 in 2014, while physicians who were not trained in emergency medicine decreased from 12,235 to 8,397.^28^,^29^ Physician assistants and nurse practitioners are increasingly prevalent among all ED clinicians, up to 14,360 in 2014.^29^ To accurately measure changes in work intensity, patient volume, patient complexity, and care intensity these changes should be benchmarked by changes in total clinician hours in the ED in future studies.

## CONCLUSION

Using survey data from a nationally representative sample of ED visits from 2007 to 2016, we found that the overall ED care intensity increased modestly as patients aged slightly, and that despite an increase in visit volume, ED visits were less likely to have low-severity illnesses. We also found that utilization of diagnostic testing, including advanced imaging, increased modestly. Furthermore, we also observed a decline in ED hospitalization rate. Future studies are needed to assess the relationship between changes in ED care intensity and the declining hospitalization rate, as well as the value of increased resource use in the ED.

## Supplementary Information



## Figures and Tables

**Table 1:** Patient and emergency department visit characteristics in National Hospital Ambulatory Care Survey, 2007 and 2016.

	2007		2016				

	Weighted Visit Count	Weighted % of total visit	Weighted Visit Count	Weighted % of total visit	Change in Weighted Visit	Change in Weighted %	
Total ED visit	116,802,066		145,591,209		28,789,143		
Patient Characteristics							
Age (in years)							
<15	22,309,924	19.1%	27,435,668	18.8%	5,125,744	−0.3%	
15–24	18,978,889	16.3%	20,674,299	14.2%	1,695,410	−2.1%	*
25–44	33,482,347	28.7%	40,013,993	27.5%	6,531,646	−1.2%	
45–64	24,493,735	21.0%	34,359,290	23.6%	9,865,555	2.6%	#
65–74	6,911,506	5.9%	10,984,887	7.6%	4,073,381	1.6%	*
75 or older	10,625,665	9.1%	12,123,071	8.3%	1,497,406	−0.8%	
Female	63,192,896	54.1%	79,594,987	54.7%	16,402,091	0.6%	
Race							
Non-Hispanic White	71,776,208	61.5%	87,940,570	60.4%	16,164,362	−1.1%	
Non-Hispanic Black	26,195,544	22.4%	30,704,146	21.1%	4,508,602	−1.3%	
Hispanic	15,803,866	13.5%	22,422,154	15.4%	6,618,288	1.9%	
Other	3,026,448	2.6%	4,524,339	3.1%	1,497,891	0.5%	
Insurance							
Private/WC/Other	42,240,378	36.2%	41,191,152	28.3%	−1,049,226	−7.9%	*
Medicare	20,130,178	17.2%	25,915,772	17.8%	5,785,594	0.6%	
Medicaid	25,920,279	22.2%	49,425,546	34.0%	23,505,267	11.8%	*
No insurance	18,026,918	15.4%	12,474,774	8.6%	−5,552,144	−6.9%	*
Unknown	10,484,313	9.0%	16,583,965	11.4%	6,099,652	2.4%	
Ambulance	18,076,808	15.5%	22,936,057	15.8%	4,859,249	0.3%	
Low-Severity Illness^1^	41,035,868	37.3%	41,593,226	30.4%	557,358	−6.9%	*
Visit characteristics							
Visit time							
Weekday	40,337,211	34.5%	52,865,496	36.3%	12,528,285	1.8%	#
Weeknight^2^	35,064,025	30.0%	41,736,017	28.7%	6,671,992	−1.4%	^
Weekend^3^	41,400,830	35.5%	50,989,695	35.0%	9,588,865	−0.4%	
Resident	9,289,073	8.0%	11,930,651	8.2%	2,641,578	0.2%	
PA/NP	15,179,703	13.0%	40,771,144	28.0%	25,591,441	15.0%	*
Region							
Northeast	20,484,250	17.5%	24,513,937	16.8%	4,029,687	−0.7%	
Midwest	25,062,048	21.5%	31,428,233	21.6%	6,366,185	0.1%	
South	48,712,961	41.7%	53,484,530	36.7%	4,771,569	−5.0%	
West	22,542,807	19.3%	36,164,508	24.8%	13,621,701	5.5%	

*PA*, physician’s assistant; *NP*, nurse practitioner; *WC*, Workers’ Compensation.

1 Compared 2007 to 2015. Unable to compare due to a change to ICD-10 coding of diagnoses without a validated crosswalk for NHAMCS, which only codes the first 4 characters of ICD-10 diagnoses.

2 Weeknights - Mon-Thursday after 5 through 8 AM the next day.

3 Weekend - Friday after 5 PM to Monday 8 AM.

* p<0.001.

# p<0.01.

^ p<0.05.

**Table 2:** Emergency department care provided, comparing 2007 and 2016 National Hospital Ambulatory Medical Care Survey.

	2007 (%)	2016 (%)	Unadjusted Relative Risk (95% CI)		Adjusted Relative Risk^1^ (95% CI)	
Advanced imaging						
CT/MRI	14.2	17.8	1.25	(1.13–1.38)	1.29	(1.17–1.41)
Ultrasound	3.0	5.2	1.73	(1.37–2.09)	1.78	(1.45–2.11)
Blood test						
Basic^2^	39.3	44.3	1.13	(1.06–1.20)	1.16	(1.10–1.22)
Special^3^	7.7	9.2	1.20	(0.94–1.47)	1.21	(0.99–1.43)
Urinalysis	22.5	26.6	1.19	(1.09–1.28)	1.22	(1.13–1.31)
Electrocardiogram	16.6	20.3	1.22	(1.09–1.35)	1.24	(1.14, 1.33)
Any radiograph	33.8	33.7	1.00	(0.94–1.06)	1.01	(0.96–1.06)
Procedures						
Urgent	14.6	10.0	0.69	(0.61–0.77)	0.67	(0.60–0.75)
Critical	0.26	0.27	1.06	(0.52–1.59)	1.06	(0.55–1.57)
Medications given in ED						
1 to 3	47.7	46.4	0.97	(0.91–1.03)	0.99	(0.93–1.05)
4 or more	6.9	15.3	2.20	(1.87–2.52)	2.17	(1.88–2.46)
Disposition						
Hospitalized	16.0	12.3	0.77	(0.64–0.90)	0.74	(0.64–0.84)
Observation	2.1	2.0	0.97	(0.49, 1.45)	0.88	(0.53–1.23)
Inpatient	13.9	10.3	0.74	(0.63–0.85)	0.72	(0.63–0.80)
Discharged	80.2	84.8	1.06	(1.03–1.09)	1.06	(1.03–1.08)

*CT*, computed tomography; *MRI*, magnetic resonance imaging; *ED*, emergency department; *CI*, confidence interval.

1 Adjusted relative risk is calculated using survey weighted multivariable logistic regression and margins post-estimation command. The model adjusted for age, sex, race, insurance status, region, ambulance use, triaged as urgent or emergent, presence of physician assistant, nurse practitioner, or resident, visit timing.

2 Include complete blood count, basic metabolic panel, liver function tests, coagulation, cardiac enzymes, blood alcohol level

3 Include human immunodeficiency virus testing, blood cultures, arterial blood gas, toxicology screening.
