# Supplementary material for: Changes in Emergency Department Care Intensity from 2007–16: Analysis of the National Hospital Ambulatory Medical Care Survey
Source: West J Emerg Med. 2020 Feb 21;21(2):209–16. doi: 10.5811/westjem.2019.10.43497 (PMC7081865; doi:10.5811/westjem.2019.10.43497)
Supplement: Supplementary file 1 [file wjem-21-209-s001.docx]

**Appendix A.** Definition for low-severity ED visit, adopted from Martsolf et al.

| **Category** | ***ICD-9* Code** |
| --- | --- |
| Skin and Soft tissue | 6800, 6801, 6802, 6803, 6804, 6805, 6806, 6807, 6808, 6809, 68100, 68101, 68102, 68110, 68111, 6819, 6820, 6821, 6822, 6823, 6824, 6825, 6826, 6827, 6828, 6829, 683, 684, 6850, 6851, 6860, 68600, 68609, 6861, 6868, 6869 |
| Contusion | 920, 9210, 9211, 9212, 9213, 9219, 9220, 9221, 9222, 9223, 92231, 92232, 92233, 9224, 9228, 9229, 92300, 92301, 92302, 92303, 92309, 92310, 92311, 92320, 92321, 9233, 9238, 9239, 92400, 92401, 92410, 92411, 92420, 92421, 9243, 9244, 9245, 9248, 9249 |
| Dental disease | 5200, 5201, 5202, 5203, 5204, 5205, 5206, 5207, 5208, 5209, 5210, 52100, 52101, 52102, 52103, 52104, 52105, 52106, 52107, 52108, 52109, 5211, 52110, 52111, 52112, 52113, 52114, 52115, 5212, 52120, 52121, 52122, 52123, 52124, 52125, 5213, 52130, 52131, 52132, 52133, 52134, 52135, 5214, 52140, 52141, 52142, 52149, 5215, 5216, 5217, 5218, 52181, 52189, 5219, 5220, 5221, 5222, 5223, 5224, 5225, 5226, 5227, 5228, 5229, 5230, 52300, 52301, 5231, 52310, 52311, 5232, 52320, 52321, 52322, 52323, 52324, 52325, 5233, 52330, 52331, 52332, 52333, 5234, 52340, 52341, 52342, 5235, 5236, 5238, 5239, 87363, 87373 |
| Dislocation/sprain | 7361, 83100, 83101, 83102, 83103, 83104, 83109, 83110, 83111, 83112, 83113, 83114, 83119, 83200, 83201, 83202, 83203, 83204, 83209, 83210, 83211, 83212, 83213, 83214, 83219, 8322, 83400, 83401, 83402, 83410, 83411, 83412, 8360, 8361, 8362, 8400, 8401, 8402, 8403, 8404, 8405, 8406, 8407, 8408, 8409, 8410, 8411, 8412, 8413, 8418, 8419, 84200, 84201, 84202, 84209, 84210, 84211, 84212, 84213, 84219, 8430, 8431, 8438, 8439, 8440, 8441, 8442, 8443, 8448, 8449, 84500, 84501, 84502, 84503, 84509, 84510, 84511, 84512, 84513, 84519, 8460, 8461, 8462, 8463, 8468, 8469, 8470, 8471, 8472, 8473, 8474, 8479, 8480, 8481, 8482, 8483, 84840, 84841, 84842, 84849, 8485, 8488, 8489 |
| Minor fracture | 8020, 80700, 80701, 80702, 80703, 80704, 80705, 80706, 80707, 80708, 80709, 8072, 8080, 8082, 80841, 80842, 80849, 8088, 8090, 81000, 81001, 81002, 81003, 81100, 81101, 81102, 81103, 81109, 81200, 81201, 81202, 81203, 81209, 81220, 81221, 81230, 81231, 81240, 81241, 81242, 81243, 81244, 81249, 81300, 81301, 81302, 81303, 81304, 81305, 81306, 81307, 81308, 81320, 81321, 81322, 81323, 81340, 81341, 81342, 81343, 81344, 81345, 81346, 81347, 81380, 81381, 81382, 81383, 81400, 81401, 81402, 81403, 81404, 81405, 81406, 81407, 81408, 81409, 81500, 81501, 81502, 81503, 81504, 81509, 81600, 81601, 81602, 81603, 8170, 8180, 8190, 8220, 82300, 82301, 82302, 82320, 82321, 82322, 82340, 82341, 82342, 82380, 82381, 82382, 8240, 8242, 8244, 8246, 8248, 8250, 82520, 82521, 82522, 82523, 82524, 82525, 82529, 8260 |
| Joint and muscle pain, tendonitis, bursitis | 71530, 71531, 71532, 71533, 71534, 71535, 71536, 71537, 71538, 71590, 71591, 71592, 71593, 71594, 71595, 71596, 71597, 71598, 71940, 71941, 71942, 71943, 71944, 71945, 71946, 71947, 71948, 71949, 7260, 72610, 72611, 72612, 72613, 72619, 7262, 72630, 72631, 72632, 72633, 72639, 7264, 7265, 72660, 72661, 72662, 72663, 72664, 72665, 72669, 72670, 72671, 72672, 72673, 72679, 7268, 72690, 72691, 72700, 72701, 72702, 72703, 72704, 72705, 72706, 72709, 7271, 7272, 7273, 72740, 72741, 72742, 72743, 72749, 72750, 72751, 72759, 72760, 72761, 72762, 72763, 72764, 72765, 72766, 72767, 72768, 72769, 72781, 72782, 72783, 72789, 7279, 72882, 72885, 7291, 7292 |
| Minor laceration | 8730, 87320, 87329, 87340, 87341, 87342, 87343, 87344, 87349, 87360, 87361, 87362, 87364, 87365, 87369, 8738, 8760, 8770, 8792, 8794, 8796, 8798, 88003, 88100, 88101, 88102, 8820, 8830, 8900, 8910, 8920, 8930, 8940 |
| Other urinary problem | 5991, 5992, 5993, 5994, 5995, 5996, 59960, 59969, 5997, 59970, 59971, 59972, 5998, 59981, 59982, 59983, 59984, 59989, 5999 |

**Appendix B. Sample STATA Code Used to Calculate Adjusted Risk Ratio**

svyset cpsum [pweight=patwt], strata(cstratm ) singleunit(centered)

svy, subpop(year0716): tab year anyscan, row

svy, subpop(year0716): logit anyscan i.year i.ager sex i.race_recode i.insurance_recode i.ambulance i.region i.resint i.pa_np i.visit_time

margins i.year, post

nlcom _b[2016.year]/_b[2007.year]
